# Geographic Object-Based Image Analysis – Towards a new paradigm

**DOI:** 10.1016/j.isprsjprs.2013.09.014

**Published:** 2014-01

**Authors:** Thomas Blaschke, Geoffrey J. Hay, Maggi Kelly, Stefan Lang, Peter Hofmann, Elisabeth Addink, Raul Queiroz Feitosa, Freek van der Meer, Harald van der Werff, Frieke van Coillie, Dirk Tiede

**Affiliations:** Department of Geoinformatics – Z_GIS, University of Salzburg, Hellbrunner Str. 34, A-5020 Salzburg, Austria

**Keywords:** GEOBIA, OBIA, GIScience, Remote sensing, Image segmentation, Image classification

## Abstract

The amount of scientific literature on (Geographic) Object-based Image Analysis – GEOBIA has been and still is sharply increasing. These approaches to analysing imagery have antecedents in earlier research on image segmentation and use GIS-like spatial analysis within classification and feature extraction approaches. This article investigates these development and its implications and asks whether or not this is a new paradigm in remote sensing and Geographic Information Science (GIScience). We first discuss several limitations of prevailing per-pixel methods when applied to high resolution images. Then we explore the paradigm concept developed by Kuhn (1962) and discuss whether GEOBIA can be regarded as a paradigm according to this definition. We crystallize core concepts of GEOBIA, including the role of objects, of ontologies and the multiplicity of scales and we discuss how these conceptual developments support important methods in remote sensing such as change detection and accuracy assessment. The ramifications of the different theoretical foundations between the *‘*per-pixel paradigm*’* and GEOBIA are analysed, as are some of the challenges along this path from pixels, to objects, to geo-intelligence. Based on several paradigm indications as defined by Kuhn and based on an analysis of peer-reviewed scientific literature we conclude that GEOBIA is a new and evolving paradigm.

## 1 Introduction

Aerial photography has a long tradition dating back to Nadar*’*s balloon-based images of Paris, France in 1858, while civilian spaceborne remote sensing (RS) began in 1972 with Landsat-1. This sensor set the standards and foundation for future multi-spectral scanner technologies and its corresponding pixel-based image analysis. Several digital classification methods (e.g., the maximum likelihood classifier) were soon developed and became the accepted processing paradigm of such imagery (Strahler et al., 1986, see also Castilla and Hay, 2008). Since the late 1990s, this “pixel-centric” view or “per-pixel approach” has increasingly been criticised (Fisher, 1997, Blaschke and Strobl, 2001, Burnett and Blaschke, 2003). The pixel based approach has been a dominant *paradigm* in remote sensing although very few scientific articles explicitly use the word “paradigm”. In fact, compared to other disciplines, remote sensing has a surprisingly small theoretical base beyond the underlying physical concepts of electromagnetic radiation and its interaction with the atmosphere and other targets. It is repeatedly argued that this focus on the pixel was and still is understandable as long as the pixel resolutions are relatively coarse, i.e., that the objects of interest are smaller than, or similar in size as the spatial resolution (Hay et al., 2001, Blaschke et al., 2004). Once the spatial resolution is finer than the typical object of interest (e.g., single trees, forest stands agricultural fields, etc.) objects are composed of many pixels and a critical question emerges: “why are we so focused on the statistical analysis of single pixels, rather than on the spatial patterns they create?” (Blaschke and Strobl, 2001).

In this article, we discuss the limitations of this *‘*per-pixel*’* approach and the rise of a new paradigm which increasingly competes with, but also complements the prevailing concept. Castilla and Hay (2008) argue that the fact that pixels do not come isolated but are knitted into an image full of spatial patterns was left out of the early *‘*per-pixel*’* paradigm. Consequently, the full *structural* parameters of the image (i.e., colour, tone, texture, pattern, shape, shadow, context, etc.) could only be exploited manually by human interpreters.

However, around the year 2000, the first commercial software appeared specifically for the delineation and analysis of *image-objects* (rather than individual pixels) from remotely sensed imagery. The subsequent area of research was referred to as *object-based image analysis* (OBIA) although terms like “object-oriented” and “object-specific” were often used (Hay et al., 1996, Hay et al., 2003, Blaschke et al., 2004). Image-objects represent *‘*meaningful*’* entities or scene components that are distinguishable in an image (e.g., a house, tree or vehicle in a 1:3000 scale colour airphoto). Thus, image-objects are inherently scale-dependent.

OBIA incorporates older segmentation concepts in an initial but essential step while further bridging spatial concepts applied to evolving image-objects and radiometric analyses that are earth surface-centric rather than biological, medical or astronomical (segmentation is also practiced in these domains). Hay and Castilla (2008) argue that Geographic space is intrinsic to this analysis, and as such, should be included in the name of the concept and, consequently, in the abbreviation: “Geographic Object-Based Image Analysis” (GEOBIA). Only then it is clear that we refer to a sub-discipline of Geographic Information Science (GIScience). While this seems both logical and obvious to Remote Sensing scientists, GIS specialist and many environmental disciplines, the fact that remote sensing images *‘*model*’* or *‘*capture*’* instances of the Earth*’*s surface may not be obvious to scientists from other disciplines such as Computer Vision, Material Sciences or Biomedical Imaging. In the remainder of this article, we will use the term “GEOBIA” henceforth.

In the following section, we will discuss the limitations under some situations of the traditional pixel-based approach. In Section 3, we analyse and discuss indications of a paradigm and discuss whether GEOBIA fulfils such criteria. In Section 4 we identify the key concepts of GEOBIA and we conclude that GEOBIA bridges remote sensing, image analysis and GIS analysis concepts.

## 2 Remote sensing and image processing concepts and limitations

The digital analysis of remotely sensed data evolved from concepts of manual image interpretation. Although developed initially based on aerial photographs, these protocols are also applicable to digital satellite imagery. Many digital image analysis methods are primarily based only on tone or colour, which is represented as a digital number (i.e., brightness value) in each pixel of the digital image (for a recent literature overview see Weng, 2009, Weng, 2011, Fonseca et al., 2009, Myint et al., 2011). Along with the advent of multi-sensor and higher spatial resolution data more research focused on *image-texture* as well as *contextual* information, which describes the association of neighbouring pixel values and has been shown to improve image classification results (Marceau et al., 1990, Hay and Niemann, 1994; 1996).

### 2.1 H- and L-resolution

In their classic paper Strahler et al. (1986) introduce a conceptual remote-sensing model comprising three sub-models: (i) the scene, (ii) the sensor and (iii) the atmosphere model. The scene is the landscape from which radiance measurements are acquired. These three sub-models together form the framework in their study, but for GEOBIA the scene and sensor/image models are particularly important. The scene model provides a simplification of the real world. It describes the real-world objects as the analyst would like to extract them from images in terms relevant to image processing. Thus, the legend is an important part of the scene model as it describes thematic characteristics of objects, and roughly implies the size of objects. Generally, more detailed thematic descriptions are related to smaller objects. For example, a forested area contains trees. The sensor model describes the specifics of the measurements from which the image is built including the number of spectral bands and their bandwidths. It also defines spatial aspects like the resolution cell, which specifies the surface area over which radiance is registered. Strahler et al. also introduced the concepts of *H*- and *L-resolution*, which, as they specifically note, should not be indicated by descriptors of *‘*High*’* and *‘*Low*’* resolution, as these are commonly applied to specific sensors and their associated pixel size [e.g. Ikonos (1.0 m PAN) vs. AVHRR (1.0 km)]. Here, (spatial) *resolution* refers to the combined spatial aspects of the scene and the sensor/image models. H-resolution indicates situations where scene objects are much larger than the resolution cells, thus several resolution cells may contain radiance data of a single object. L-resolution represents the opposite situations where scene objects are much smaller than the resolution cells. While a pixel contains both H- and L- resolution information, each of which can be used for image analysis (Hay et al., 2001) GEOBIA is primarily applied to very high resolution (VHR) images, where image-objects are visually composed of many pixels; and where it is possible to visually validate such image-objects (i.e. H-resolution case). The use of GEOBIA, however, is not limited to images with small resolution cells. If the legend of the scene model is generalized, i.e. a higher hierarchical level of the legend is applied, then the size of scene objects will increase and an L-resolution situation may turn into an H-resolution situation.

A common issue with coarse resolution cells is that they combine spectral properties of heterogeneous land cover. For example, in the case of a resolution cell of 1 km*^2^* in a forested area, the scene will contain mostly forest (typically of more than one species), but probably also open patches, paths and roads, or small fens etc. Although the spectral properties will be dominated by forest vegetation, they will not represent *‘*pure*’* forest. Hence, spectral mixing increases in images with coarser resolution cells which in turn leads to confusion during classification. While creating object attributes, the spectral properties of individual cells are averaged for the entire object. This reduces classification confusion as averaging diminishes the (within-object) variance and seems to be appropriate for classification of coarse resolution images. At present, per pixel image analysis of coarse spatial resolution images (e.g., MODIS, AVHRR) remains the base producer of spatially continuous land cover information. The production of classified thematic maps by broadband multi-spectral imagery, however, has evolved due to the advent of high spatial resolution imagers.

### 2.2 Advances in image classification

Throughout the last 15–20 years, advanced classification approaches, such as artificial neural networks, fuzzy logic/fuzzy-sets, and expert systems, have become widely applied for image classification. Weng (2009) provides a valuable list of the major advanced classification approaches that have appeared in recent literature, dividing the approaches into the following major categories with subsequent sub-categories: *per-pixel* (17 categories), *sub-pixel* (7 categories), *per-field* (6 categories), *contextual based approaches* (13 categories), *knowledge based* (6 categories), and *combinational approaches* of multiple classifiers (14 categories). Weng (2009) includes GEOBIA within the category *‘Per-field* classification*’* (see next paragraph), which may be used to explain the role of segmentation in GEOBIA: segmentation is only one possible means to delineate objects of interest. If they are derived otherwise, e.g. imported from a GIS database, we may more explicitly call the subsequent classification process a per-field classification. Interestingly, GEOBIA methods are only one of the 63 specified by Weng, although its number of literature references per category (from international journals between 2003 and 2004) is the highest overall.

In an effort to improve pixel based classifications by exploiting scene characteristics other than *‘*colour*’* – such as tone, shape pattern, context etc., the most widespread approaches incorporate information on image-texture and pattern, based on moving window or kernel methods, the most common being the Grey Level Co-occurrence Method (GLCM) (Haralick et al., 1973, Marceau et al., 1990). Since the late 1980s, geostatistical approaches have also been used to exploit the information content of remote sensing imagery, in particular variogram-based approaches. The variogram is a measure of spatial dependence, that has been used to quantify image structure linking remote sensing and geostatistical theory (Curran, 1988). It has also been proposed as an alternative measure of image-texture as it relates to image variance and spatial association (Hay et al., 1996). For the sake of completeness we note that we leave out sub-pixel classification in this brief discussion since we concentrate on H-Res situations.

Per-field classification approaches have shown improved results in some older studies (e.g. Lobo et al., 1996). In fact, the widely-known ECHO algorithm (Kettig and Landgrebe, 1976) is a two-step approach using the results from an initial single pass region growing segmentation as outlines for a subsequent *‘*per-field classification*’*. Results of per-field classifications are often easier to interpret than those of a per-pixel classification (Blaschke et al., 2004). The results of the latter often appear speckled even if post-classification smoothing is applied. *‘*Field” or *‘*parcel*’* refers to homogenous patches of land (agricultural fields, gardens, urban structures or roads) which already exist and are superimposed on the image.

### 2.3 Limitations of the *‘*per-pixel*’* approach

Most of the methods for image processing developed since the early 1970s are based on classifications of individual pixels utilizing the concept of a multi-dimensional feature space. In Section 2.2 we have shown that a range of sophisticated and well established techniques have been developed that classify L-resolution images by pixels. However, it is increasingly recognized that the current demand from the remote sensing community and their clients – in respect to ever faster and more accurate classification results – is not fully met due to different characteristics in high resolution imagery and varying user needs (see e.g. Wang et al., 2009). New H-resolution sensors significantly increase the *within-class* spectral variability and, therefore, decrease the potential accuracy of a purely pixel-based approach to classification. Hay et al. (1996) referrs to this as ‘The H-Resolution problem’.

### 2.4 Challenge 1: objects

Objects are never exclusively a construct used to discuss environments as such; instead they are part of discourses that shape our thinking about space, time and relations (Massey, 1999). The key point is that pixels may not be seen relationally. However, objects are both the product of the attention of a thoughtful observer, and the resulting matter and processes. Objects may also be the product of the representational devices deployed (Ahlqvist et al., 2005) – that is, the emergent scene structures/patterns resulting from specific processes *‘*captured*’* at a particular scale (spatial, spectral, temporal, radiometric). Mixed pixels may serve as an illustration here: a pixel whose digital number represents the average of several spectral classes within the area that it covers on the ground, each emitted or reflected by a different type of material are likely to be misclassified and their existence is highly influenced by the resulting variations caused by the data acquisition process. In contrast, GEOBIA is focused on research into the conceptual modelling and representation of spatially referenced imagery. By bridging GIS, remote sensing and image processing it integrates numerous *‘*spatial perspectives*’*. For example, it relies on the concepts of space, spatial features and geographical phenomena, and it provides a spatial view into various kinds of physical and abstract information objects including natural and anthropogenic landforms/landcover and the cultures that may have formed them, e.g., Quebec*’*s agricultural long-lots, rice terraces in China, or favela*’*s in Brazil.

### 2.5 Challenge 2: shape

Identification of objects by human vision is based on a combination of factors like shape, size, pattern, tone, texture, shadows and association (Olson, 1960). Geometry, the combination of shape and size, together with tone are major factors. Shape refers to general form or outline of individual objects, while tone indicates the spectral properties of an individual band (Lillesand et al., 2008). With per-pixel classification, spectral properties are by far the most important for identifying objects; however, by applying filters, some local variance in pixel values can also be included, though spectral information is dominant. When an object class has a unique spectral *‘*signature*’*, classification is relatively *‘*trivial*’*. However, when an object class shares spectral signatures with other classes, classification often proves difficult. We note that an implicit shape is seldom if ever evaluated or defined pre-classification – except in the case of feature detection (and template matching).

**Fig. 1:**
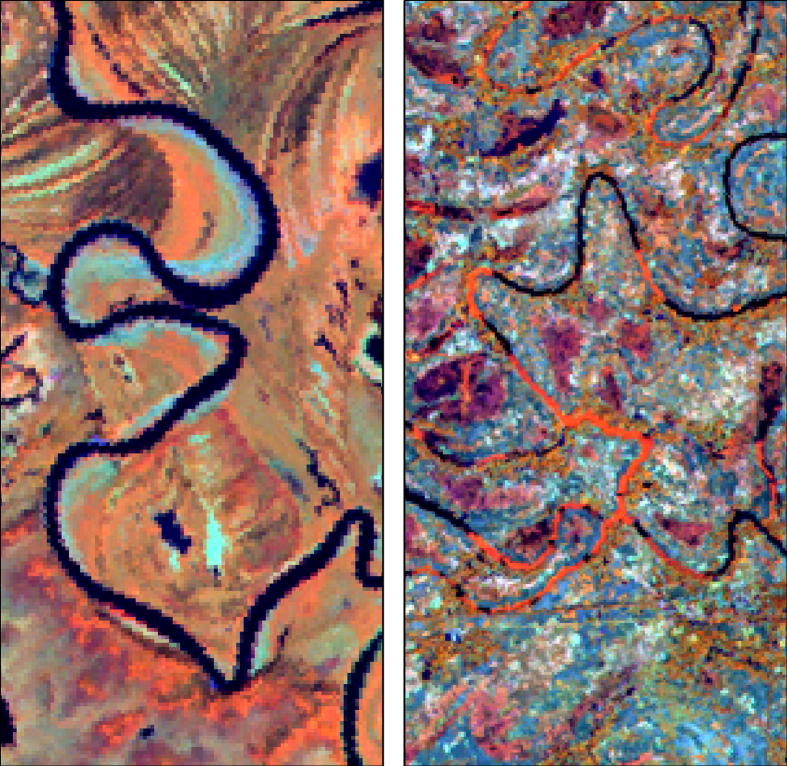
Subsets of Landsat TM scenes from Alaska (left) and Bangladesh (right). The left water filled channel intermingles with an old sediment-filled channel. The right portion of the water filled channel is overgrown by vegetation.

GEOBIA offers possibilities for situations where spectral properties are not unique, but where shape or neighbourhood relations are distinct. For example, river meanders will have the spectral properties of water when they are still active, but once they are abandoned a range of possibilities exists (Addink and Kleinhans, 2008). They can remain water filled, they can be filled in by sediment, they can be overgrown by vegetation, or a combination of these three land cover situations might occur (Fig. 1). These land-cover types are not unique to meanders, thus prohibiting their identification by spectral properties alone. However, the shape of the meander will remain unchanged, thus offering a unique property that can be used to identify meanders independent from their land cover appearance.

The size of the meanders depends on the discharge and may therefore show considerable variation. By creating object sets by different spectral heterogeneity thresholds and adapting the shape criteria, meanders with different sizes and different spectral properties could be identified. Although geometry will often not be distinct by itself, in many situations it will be a valuable factor in the identification of objects.

### 2.6 Challenge 3: texture

In natural or near-natural environments, transitions may by fuzzy or gradient-like. This causes problems for a classification process that necessitates crisp decisions. A gradient operator applied to a raw intensity image will not only respond to intensity boundaries but also the intensity variation due to object texture resulting in a significant number of false positives. *Image-texture* refers to particular frequencies of change in tones and their resulting spatial arrangements (Hay and Niemann, 1994). For the human interpretation of images the visual impression of *smoothness* or *roughness* of an area is an important cue. For example, water bodies typically are finely (smoothly) textured; grass may be regarded as a medium texture and brush as rough (although there are always exceptions). As these simplified examples reveal, image-texture involves spatial context; thus it is unable to exist at a single pixel or point (Hay et al., 1996). In the per-pixel approach, information on texture is typically derived using a moving window or kernel method of a fixed size, shape and (limited) orientation(s). A more powerful form of image-texture is to build on the spatial interaction of neighbouring image-objects (Hay and Niemann, 1994, Powers et al., 2012).

### 2.7 Challenge 4: context and pattern

**Fig. 2:**
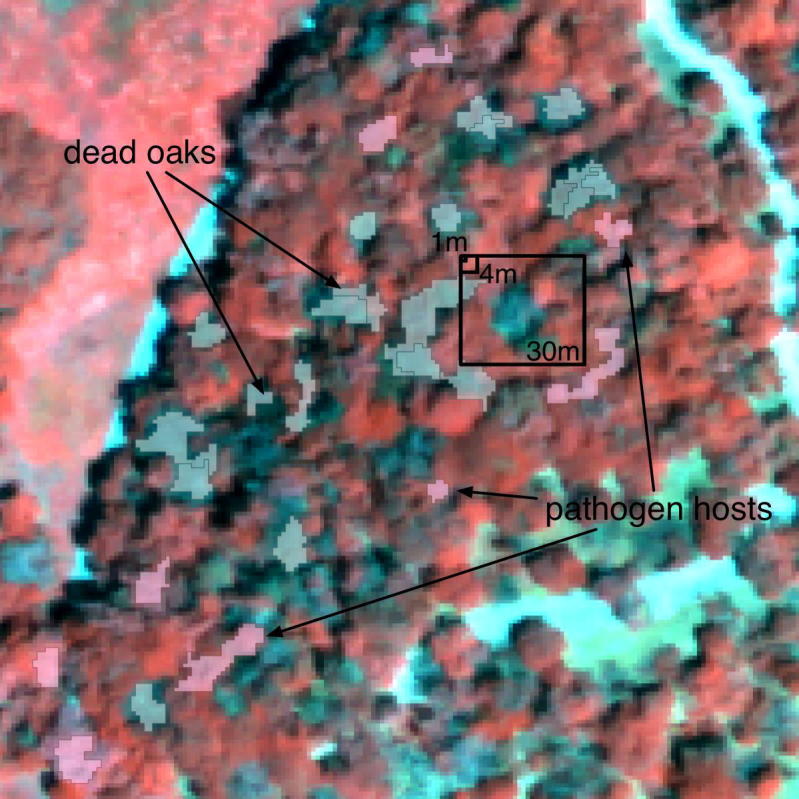
False-colour digital image of a forest stand with sudden oak death in CA showing selected objects representing dead trees (grey) and associated hosts (magenta), and illustrating three common image spatial resolutions: 30 m, 4 m and 1 m. (For interpretation of the references to colour in this figure legend, the reader is referred to the web version of this article.).

Addressing real entities such as trees may require to mining their context and pattern across scales. One convincing example where ecological information could be addressed through multi-scale object building is provided by de Chant and Kelly (2009). For a new forest disease in California (USA) called *sudden oak death*, these authors found key insights into disease ecology and impact by considering individual trees as objects in a remote sensing classification process. They confirm the importance of non-oak hosts in spreading the disease by examining the spatial patterning of individual oaks and their neighbours in space and time (Kelly et al., 2008, Liu et al., 2006); and that these characteristics are relevant at multiple scales, and displayed hierarchies (Liu et al., 2007). Even sophisticated adaptive kernel-based methods would fail. Additionally, these kinds of multi-scaled patterns can be used to construct rules for classifying image-objects and refining GEOBIA classification results (Liu et al., 2008), and in concert with other key concepts (e.g. shape, texture, etc.) convey important agency to the resulting objects (see Fig. 2). Such *‘*rules*’* may also be used in Cellular Automata and Agent based modelling (Marceau and Benenson, 2011).

### 2.8 Challenge 5: semantics and knowledge integration

Basic entities composed of pixels are limited to be used as constituents for semantic information. Pixels are limited in supplementing our implicit knowledge with explicit knowledge obtained from formal learning situations (e.g. spectral behaviour of stressed vegetation). From an Artificial Intelligence (AI) perspective knowledge can be distinguished as procedural and structural knowledge. Procedural knowledge is concerned with specific computational functions and can be represented by a set of rules. Structural knowledge – understood here as declarative knowledge – implies how concepts of a domain are interrelated: in our case this means, the relationship between image-objects and *‘*real world*’* geographical features (Castilla and Hay, 2008). Structure is characterised by high semantic content and therefore is more difficult to tackle. Tiede et al., 2010a, Tiede et al., 2010b applied semantic modelling to deliver functional spatial units (so called biotope complexes) for regional planning tasks. The categories addressed (e.g. mixed arable land, consisting of different types of agricultural fields in a specific composition) represent composite objects consisting of homogenous building blocks (elementary units). The target categories are modelled by their specific internal arrangements. This internal arrangement is a structural, not a statistical (i.e. pattern) feature and requires explicit spatial relationships to be addressed. To underline this fact, the term *‘*class modelling*’* is used by Lang (2008).

Structural knowledge can be organised into knowledge organizing systems, realised by graphic notations such as semantic networks (Liedtke et al., 1997, Sowa, 1999), and by more mathematical theories like the formal concept analysis (FCA, Ganter and Wille, 1996). Within image analysis, semantic nets and frames (Sowa, 1999) offer a formal framework for semantic knowledge representation using an inheritance concept (is part of, is more specific than, is instance of) (Lang, 2005; 2008) – which is also a foundation of Object-Oriented (OO) programming.

## 3 Is GEOBIA a paradigm?

### 3.1 Kuhn*’*s paradigm concept

In 1962 Thomas Kuhn published “*Structure of Scientific Revolutions*”, a highly influential book that described the process of intellectual revolution. The key concept – if extremely condensed and simplified – is that common practice may be regarded as normal science whereas new concepts when clearly contradicting established thoughts may be called revolutionary science. Kuhn*’*s main hypothesis is that scientific development is not smooth and linear. Instead it is episodic – that is, different kinds of science occur at different times. The most significant episodes in the development of a science are *normal science* and *revolutionary science*. It is not a cumulative process, since revolutionary science typically discards some of the achievements of earlier scientists. Typically, individual scientists seek to solve the puzzles they happen to be faced with and they are not interested in a fixed scientific method per se. Instead scientists make discoveries based on their training with exemplary solutions to past puzzles, which Kuhn calls *paradigms*. There is some vagueness in the definition of a paradigm (see Kuhn, 1962, p. 181ff). Nonetheless, Kuhn provides a widely accepted framework for describing how change, and all that implies, occurs in science. A paradigm is “what the members of a scientific community share” (Kuhn 1962, p. 176). This comprises not only the laws and results of this scientific community but the methodologies, the aims, the conventions, the research questions and their unsolved problems. Research questions are expected to be answered within the constraints of the paradigm.

Who is this scientific community? It includes the scientists, students, observers and philosophers who share and strive to maintain the paradigm. The community changes as scientists are trained and die; the paradigm changes as new observations are made. However the change is such that the paradigm will be strengthened since it is used as an exemplar. Thus, the paradigm notion refers to a shared set of assumptions, values and concepts within a community (Pollack, 2007). Additionally, according to Kuhn, a scientific revolution will revise some of the previous paradigm but not necessarily all of it. To be accepted, a proposed new paradigm must retain some achievements of its predecessor; as well as scientists trained in the old paradigm.

### 3.2 What characterizes a paradigm?

Social scientists have adopted Kuhn*’*s concept of a paradigm shift to denote a change in how a scientific community organises and understands reality. A *dominant paradigm* refers to the values, or system of thought in a society that are most standard and widely held at a given time. Furthermore, dominant paradigms are typically shaped by the community*’*s cultural background and their historical development. Some authors have commented in previous writings that GEOBIA is a paradigm (e.g. Hay and Castilla, 2008). However, it should be stated explicitly here that we do not claim that the per-pixel remote sensing approach is wrong, merely that we now have a different understanding of the world. For over a decade this has been supported by numerous journal articles that have shown that object-based classification results (especially of H-resolution imagery) are consistently better than those based on traditional pixel-based approaches (e.g., Shackelford and Davis, 2003, Yu et al., 2006, Blaschke, 2010, Myint et al., 2011, Whiteside et al., 2011).

We also note that the concept and act of *revolution* that Kuhn describes was necessary (especially in earlier times) for change(s) to take place, as the scientific establishment was typically very conservative, and its power-base, which was often associated with important social, political and financial structures, was held in the hands of a select few influential individuals – whom seldom relinquished it without a fight (a.k.a revolution). However today, ubiquitous global media and communication technologies increasingly place the power of change in the hands of *‘*the people*’* rather than a select few. And though *‘disruptive technologies* do exist, and ideas can quickly become *‘*viral’, the vast majority of today’s scientific change follows a more evolutionary path, rather than that of a revolution (Hay and Castilla, 2008).

In fact, Kuhn also claims that the world has changed as he noted “...we may want to say that after a revolution, scientists are responding to a different world” (Kuhn 1962, p. 111). And, while Kuhn states that the new paradigm replaces the old one, dozens of scientists from different disciplines have more recently argued that some disciplines are “multi-paradigmatic” (e.g. Lukka, 2010) and that the diversity of world views is the key to interpretation and understanding of it.

Based on a synthesis of these ideas, combined with our own experiences, we suggest the following general conditions support the idea of a (new) paradigm becoming more widespread or even accepted:

• The absolute number of (new paradigm) publications increases along with the acceptance rate in renowned journals.
• Conferences devoted to discussing ideas and methods central to the (new) paradigm.
• The development of professional organizations that give legitimacy to the (new) paradigm.
• Dynamic leaders who introduce and purport the (new) paradigm through papers, presentations, and more recently through blogs, tweets, Wiki*’*, etc.
• Books and special issues of journals on the new approach/paradigm.
• Scholars who promulgate the paradigm*’*s ideas by teaching it to students and professionals.
• The creation of related free and open source software/tools and online communities to support the use, development, and promotion of these new ideas and methods.
• The development of commercial software and promotion of industry supported communities and programs.
• The creation and implementation of new (related) standards, and their (continued) evolution.
• Government agencies who give credence to the paradigm, informally or formally.
• Increased media coverage.

Although this list is not exhaustive and some parameters are not easily measurable, we will provide support in the following sub-section that GEOBIA can be regarded as a paradigm.

### 3.3 Facts which support the GEOBIA paradigm hypothesis

Synthesizing existing definitions (Hay and Castilla, 2008, Hay and Blaschke, 2010) we may state that GEOBIA is a ‘recent’ approach (including theory, methods, and tools) to partition remote sensing imagery into meaningful image-objects, and assess their characteristics through scale. Its primary objective is the generation of geographic information (in GIS-ready format) from which new spatial knowledge or “geo-intelligence” (Hay and Castilla, 2008) can be obtained. Here, geo-intelligence is defined as *geospatial content in context* (Hay and Blaschke, 2010). GEOBIA is not limited to the remote sensing community but also embraces GIS, landscape ecology and GIScience concepts and principles, among others. The following points support the notion that GEOBIA is a new paradigm.

Blaschke (2010) previously diagnosed a significant body of relevant literature in this field and noted a particularly fast increase in peer-reviewed literature. Similarly, we undertook a brief literature survey using Google Scholar (GS), WebofKnowledge (WoK) and SCOPUS (Elsevier). Results show that not only is the number of articles increasing, but that the rate of growth is dramatically accelerating. Blaschke (2010) performed his search in April 2009 and identified 145 journal papers relevant to GEOBIA. Since more and more GEOBIA methods are integrated into application papers it is difficult to provide an exact number. However, based on a literature analysis using Web of Knowledge and Scopus by using various spelling alternatives we estimate the number of relevant journal articles to be over 600 (September 2013) which means that they have more than quadrupled over the last four and a halfyears (see Table 1).

**Table 1:** Citations of highly cited GEOBIA papers in Web of Knowledge (WoK), SCOPUS and Google Scholar (GS) for September 2013 compared to the respective figures – if available – from Blaschke (2010) based on a survey conducted in April 2009.

2013				2009	
Authors	WoK	SCOPUS	GS	WoK	GS
Benz et al. (2004)	570	655	1139	150	220
Blaschke (2010)	217	338	555	–	–
Blaschke et al. (2000)	–	–	250	–	76
Blaschke and Strobl (2001)	–	172	383	–	–
Burnett and Blaschke (2003)	179	182	335	63	101
Desclée et al. (2006)	106	125	164	–	–
Câmara et al. (1996)	168	182	857	49	331
Yu et al. (2006)	159	163	273	–	–
Shackelford and Davis (2003)	100	126	201	–	–
Hay et al. (2001)	88	87	162	–	–
Hay et al. (2003)	104	134	229	41	71
Hay et al. (2005)	102	117	160	–	–
Laliberte et al. (2004)	155	162	255	–	–
Walter (2004)	143	185	292	–	–

*For comparison: top image processing articles beyond remote sensing applications*					
Haralick and Shapiro (1985)	889	812	2134	720	1104
Pal and Pal (1993)	1120	1362	2679	777	1187

Several international journals dedicated special issues to GEOBIA including *GIS – Zeitschrift für Geoinformationssysteme* (2001), *Photogrammetric Engineering & Remote Sensing* (2010; 2014), *Journal of Spatial Science* (2010), *Remote Sensing* (2011; 2014), *Journal of Applied Earth Observation and Geoinformation* (2012), *IEEE Geoscience and Remote Sensing Letters* (2012).

Several workshops and four bi-annual international conferences were held including (i) the *OBIA International conference*, Salzburg, July 2006, Austria; (ii) *Object-based Geographic Information Extraction*, June 2007, Berkeley, USA; (iii) *GEOBIA 2008: Pixels, Objects, Intelligence: Geographic Object-based Image Analysis for the 21st Century*, Calgary, Canada; (iv) *Object-based Landscape Analysis*, 2009, Nottingham, UK; (v) *GEOBIA 2010*, Ghent, Belgium, (vi) *GEOBIA 2012*, May 2012, Rio de Janeiro, and (vii) GEOBIA 2014, Thessaloniki, Greece.

Several books are dedicated to OBIA and GEOBIA topics, most notably the Springer book edited by Blaschke et al. (2008). According to information provided by Springer (personal communication: 04 June 2012) the total number of chapter downloads is 20033.

Other indications mentioned above include respective university classes, job announcements or even dedicated professor positions. We could not carry out an in-depth survey but we are aware of individual evidence at Universities in Europe, the US, Canada, Brazil, Australia and China. A rough internet-based search finds more than 30 finished or on-going PhD projects which have the terms OBIA or GEOBIA included in the title, keywords or abstract.

Last but not least we may mention that a significant amount of public institutions and agencies are using GEOBIA software at professional and operational levels. This ranges from Nature Conservation agencies to the Military which creates a high demand in training and education beyond simple software competency.

## 4 Geographic Object-based Image Analysis – key concepts

### 4.1 Human interpretation and perception as guiding principles for GEOBIA

In an effort to better understand and develop a more explicit GEOBIA framework, Hay and Castilla (2008) provided a number of *tenants* or fundamental guiding principles. They described GEOBIA as exhibiting the following core capabilities: (i) data are earth (Geo) centric, (ii) its analytical methods are multi-source capable, (iii) geo-object-based delineation is a pre-requisite, (iv) its methods are contextual, allowing for ‘surrounding’ information and attributes, and (v) it is highly customizable or adaptive allowing for the inclusion of human semantics and hierarchical networks. Lang (2008) also describes a selection of GEOBIA guiding principle for complex scene content so that the imaged reality is best described, and the maximum (respective) content is understood, extracted and conveyed to users (including researchers). For details see Lang (2008, pp. 14–16).

Many consider that the ultimate benchmark of GEOBIA is the generation of results equalling or better than human perception, which is far from trivial to numerically quantify and emulate. Human perception is a complex matter of filtering relevant signals from noise (Lang, 2008), a selective processing of detailed information and, finally, experience. Enormous advances have been made in computer vision but the potential of human vision remains to be achieved. While biophysical principles like retinal structure and functioning and singular processes such as the cerebral reaction are analytically known, we still lack the bigger ‘picture’ of human perception as a whole (Lang, 2005). Cognitive psychology tells us about mechanisms we use in perceiving patterns and spatial arrangements, and Marr (1982) provides a conceptual framework of a three-levelled structure of visual information processing (Eysenck and Keane, 1995).

Lang (2005) elaborates on the relation between image perception and image interpretation and refers to the original literature in neuro-psychology for concepts such as ‘experience’ in the context of images and suggests that more than one model is used to construct meaning from an image (Lang et al., 2004). Image interpretation, when dealing with an unfamiliar perspective and scale, requires *‘*multi-object recognition*’* in a rather abstracted mode, and the interpreter needs to understand the whole scene. According to Gibson (1979), values and meanings of objects are attributed via (object) *‘*affordance*’* (Lang et al., 2009). The skilled visual interpreter may recognise some features instantly and others by matching the visual impression against experience or examples listed in an interpretation key. All these concepts – and many which cannot be discussed here – are difficult to be used in per pixel analyses. However, they can be addressed more appropriately through GEOBIA concepts, of which several key components will be discussed in the following section.

Blaschke et al. (2004) elucidate the relationship between pixels and image-objects. A pixel is normally the smallest entity of RS imagery. A pixel*’*s dimensions are determined by the sensor and scene geometric models. Image-objects as defined by Hay et al. (2001) are basic entities, located within an image that are perceptually generated from High-resolution pixel groups, where each pixel group is composed of similar data values, and possesses an intrinsic size, shape, and geographic relationship with the real-world scene component it models. Possible strategies to model spatial relationships and dependencies present in RS imagery are centred on image segmentation and *‘*objectification*’* whereby the latter is understood as the integrated spatial and thematic object definition. The following key concepts, while not exhaustive represent important building blocks of GEOBIA methodology.

### 4.2 Image segmentation: not an end in itself

In GEOBIA, image segmentation is not an end in itself. Segmentation is the partitioning of an array of measurements on the basis of homogeneity. It divides an image – or any raster or point data – into spatially continuous, disjoint and homogeneous regions referred to as *‘segments’*. In feature extraction, it can be regarded as an end in itself. In GEOBIA, it is one step in a processing chain to ultimately derive *‘*meaningful objects*’*. Blaschke et al. (2004) following the basic methodology of Burnett and Blaschke (2003) describe this process when referring to the *‘*near-decomposability*’* of natural systems as laid out by Koestler (1967). Simply speaking, the resulting internal heterogeneity of a segment under consideration shall be less than the heterogeneity when it is taken in conjunction with its neighbours.

Image segmentation was well established throughout the late 1970s and the 1980s (see Haralick and Shapiro, 1985), with numerous segmentation algorithms available (Pal and Pal, 1993). Traditional segmentation methods are commonly divided into three main approaches: (i) pixel-, (ii) edge and (iii) region-based segmentation methods. Though available to the computer science community, image segmentation was seldom used (exceptions were already acknowledged, see e.g. Kettig and Landgrebe, 1976, Câmara et al., 1996) for the classification of earth observation data, as most algorithms were developed either for pattern analysis, the delineation of discontinuities on materials or artificial surfaces, or quality control of products (Blaschke et al., 2004).

While GEOBIA may be believed to be critically dependent on the appropriate choice of a segmentation technique there are very recent developments which are decreasingly dependent on the initial segmentation. In several of these approaches (see e.g. Baatz and Schäpe, 2000, Lang et al., 2010, Tiede et al., 2010a, Tiede et al., 2010b, 2011, 2012) segmentations are used very flexibly in initial stages and are also tailored in a later stage for specific classes or regions in the image when the classification process requires this. A group of researchers from Brazil recently developed segmentation software in which different shape features may be used to express heterogeneity within the region growing process (Feitosa et al., 2011). In their experiments they “optimize” the segmentation parameter values for each set of shape features being considered. Their results showed that the segmentation accuracy may be considerably improved when shape features are used in the formulation of a heterogeneity criterion – which is essential for a *‘*meaningful*’* segmentation.

### 4.3 Putting pixels into context

**Fig. 3:**
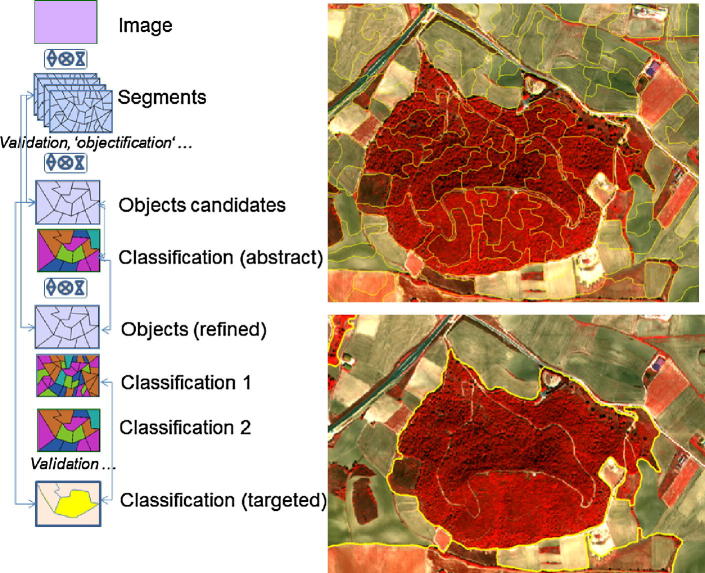
Idealized GEOBIA workflow that illustrates the iterative nature of the object building and classification process which incorporates GIScience concepts.

A key issue when segmenting earth observation data is the fundamental difference between a scene and an image. An image is a collection of measurements (*at a specific time and location*) *from* a sensor that are arrayed in a systematic fashion. Thus a scene-object (for all intents and purposes) is a (fiat) real world object, while an image is a collection of spatially arranged samples that model the scene. Essentially, it is the sensor*’*s *‘*view*’* of the scene. Consequently, while image segmentation groups pixels that are alike in terms of registered values, it is possible and highly probable that a one to one relationship may not exist between scene objects and the image objects or the underlying segments that model them (see Fig. 3 and explanation below).

Another issue is that images are only snapshots, and their size and shape are dependent upon the sensor type and spatial sampling of the remotely sensed image from which they are derived. Thus, segments are not by definition *‘*meaningful*’*. For example, through segmentation, two adjacent forest stands could end up in a single (image-) object even though they are managed differently or are owned by different proprietors.

Fig. 3 exemplifies a complex workflow from ‘segments’ to image-objects. The latter are *‘*meaningful*’* groupings with regard to a particular context or aim. Imagine the existence of a real boundary between two forest stands, such as a creek. This boundary would also need to be spatially and spectrally distinct in relation to the spatial resolution of the image in order for a segmentation process to generate a new object. Conversely, (at a finer spatial resolution) a single forest stand can feature considerable internal variability (e.g., due to the health condition of the constituent trees) causing the segmentation process to over-segment the scene and create multiple objects within a single stand.

**Fig. 4:**
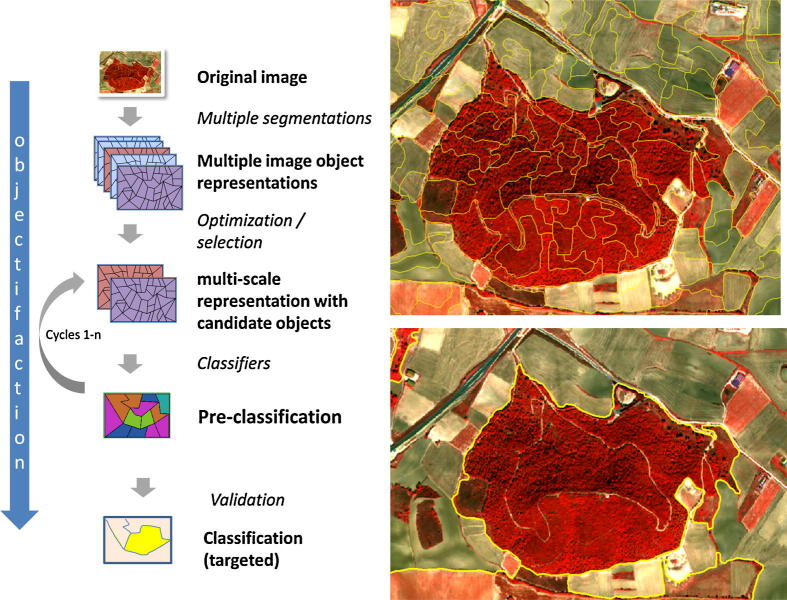
Pixels and image-objects as information carriers: constant size, constant shape and implicit location vs. unique area/outline information derivatives and statistical descriptors of the interior. For the sake of simplicity, the temporal dimension is left out here.

A final thought pertaining to the configuration at which image-objects manifest themselves. Due to the typical pixel-wise representation of earth observation data, segmentation of image data always yields *‘*pixelated*’* object shapes. This could cause problems when comparing these image-objects with other spatial information like e.g. cartographic data. Segmentation of earth observation data is very often followed by an *‘*objectification*’* (i.e. classification) step eventually leading towards meaningful object definitions. Still, a core concept is that objects are the main information carrier (see Fig. 4).

Among open challenges in GEOBIA are methods describing multi-temporal behaviour (De Chant and Kelly, 2009, Chen et al., 2012). Two recent papers describe additional approaches for cascaded multi-temporal classifications pursued at the Catholic University Rio de Janeiro (Feitosa et al., 2011, Leite et al., 2011).

### 4.4 GEOBIA and the Object-Oriented (OO) data model

The subject of GEOBIA is related to concepts of object-oriented software and to object handling in the GIS world; for additional information the reader is referred to an OO review paper by Bian (2007). OO concepts and methods have been successfully applied to many different problem domains, and there is great opportunity to adapt and integrate many of its beneficial components to GEOBIA (Hay and Castilla, 2008), as the majority of early GEOBIA research was conducted without OO software, tools or languages. This integration not only includes OO programming, but all the corpus of methods and techniques customarily used in biomedical imaging and computer vision (among others) that remain mostly unknown to the majority of the remote sensing community.

Unlike the geo-relational data model, which separates spatial and attribute data and links them by using a common identifier, the object-oriented data model views the real world as a set of individual objects that may have spatial and non-spatial interrelationships among each other. Thus, an object has a set of properties and can perform operations on requests (Worboys, 1995).

**Fig. 5:**
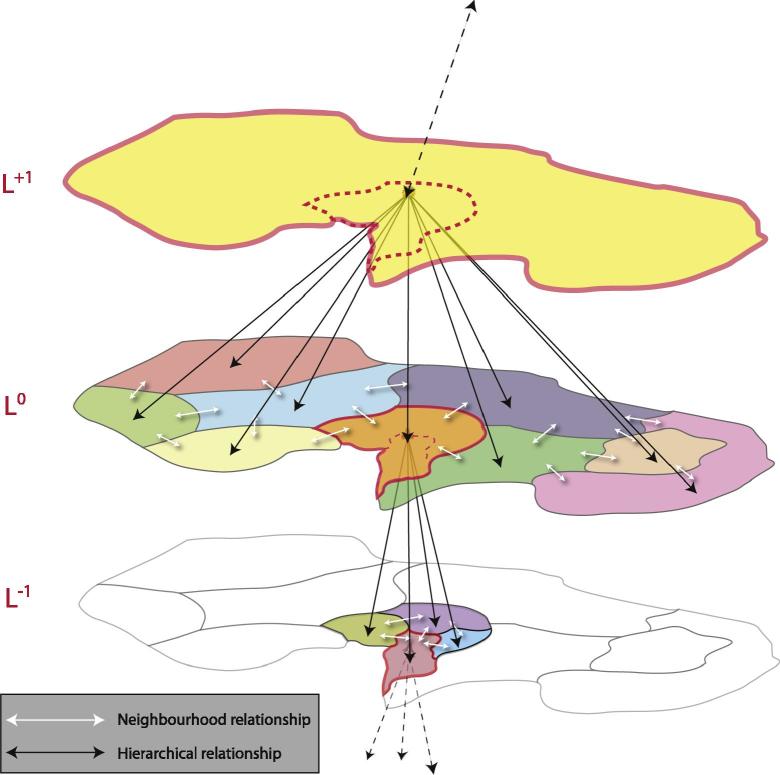
Hierarchy of image objects. Objects have (topological) neighbourhood relationships and have hierarchical relationships, such as “is-part-of” or “consists-of”. Respectively they can be (nearly) decomposed.

Baatz et al. (2008) argue to call more complex GEOBIA workflows “object-oriented,” due to the fact that the objects are not only used as information carriers but are modelled with the continuous extraction and accumulation of expert knowledge. That is, by incorporating expert knowledge from the application and image processing domain the initial segmentation results are optimized step by step through dedicated processing steps. The aim of this optimisation process is to generate image-objects that fulfil the major criteria of the intended entities in the image domain. In many cases, an *a priori* defined ontology of the image-objects to detect is used as a tool to model real-world objects (Clouard et al., 2010, Hofmann et al., 2008, Hofmann et al., 2006). In order to describe natural variability, many models need to be capable of expressing their vagueness (e.g. by fuzzy rules) and to be adaptable according to unforeseeable imaging situations (Benz et al., 2004, Hofmann et al., 2011). For instance, a meadow can be spectrally more or less homogeneous but at the time of the image acquisition some agricultural machines could be left there. Rules which refer the larger entity of a meadow consisting of thousands of image pixels should allow for small islands of contrast within. This would typically be handled through object-sub-object relationships (see Fig. 5).

### 4.5 GIS-like functionality for classification

When classifying segments – rather than pixels – size, shape, relative/absolute location, boundary conditions and topological relationships can be used within the classification process in addition to their associated spectral information (as done by human photo interpreters). In fact, some GEOBIA researchers claim that this is a key to the popularity and utility of this approach. There is increasing awareness that object-based methods make better use of – often neglected – spatial information implicit within RS images, which ultimately allows for a tightly coupled or even full integration with both vector and raster based GIS. In fact, when studying the early GEOBIA literature it may be concluded that many applications were driven by the demand for classifications which incorporate structural and functional aspects.

### 4.6 Multi-scale and hierarchies

A very important concept to distinguish GEOBIA from per-pixel approaches is the ability to address a multiplicity of scales within one image and across several images. Since its inception, the discipline of Ecology has considered the notion of scale domains and scales of variability of different ecological factors, such as plant morphology and soil nutrients (Greig-Smith, 1979). In fact, this concept is used to facilitate the search for underlying patterns and mechanisms and it may be claimed that the concurrence of scales (often achieved through different levels of segmentation) may be a seen as a way to model relatively continuous phenomena (Allen and Starr, 1982) – though Bian (2007) notes the challenges of delineating the edge(s) of such phenomena. One may critically note that GEOBIA methods face difficulties in environmental gradients where parameters gradually, but continuously change. However, there are many examples in nature where the effects of *‘*processes*’* are not truly continuous, and as such, GEOBIA may address a more or less seamless transition between two stages through super-object/sub-object relations. If a transition was really continuous then the *field concept* (Cova and Goodchild, 2002) may be an appropriate conceptual metaphor to qualify it, though we have not seen it implemented in existing software. The term “field” here is completely different from the “per-field” classification concept mentioned earlier. The latter refers to fields in a sense of parcels. As such, the *field and object-based* approaches to spatial data modelling are not mutually exclusive (Worboys 1995, p. 177). In fact, the concept of Cova and Goodchild (2002) is a hybrid concept of object fields in which every point in geographic space is mapped not to a value but to an entire discrete object.

**Fig. 6:**
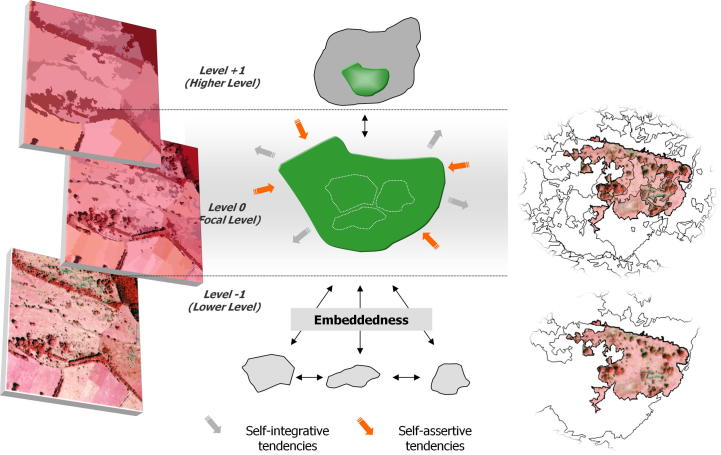
Conceptual illustration of a multi-scale representation of an imaged landscape according to hierarchy theory principles. Object generation on the scale level of concern (*‘*focal level*’*) is embedded in higher level objects and lower level ones. Note that within GEOBIA, higher hierarchical levels usually correspond to increasing average object size. Objects have both self-integrative (*‘*part-of …*’*) and self-assertive (*‘*aggregates of …*’*) tendencies, and thereby feature the basic characteristics of holons. From Lang et al. (2004).

Burnett and Blaschke (2003) developed a five step methodology which they called “multi-scale segmentation/object relationship modelling” (MSS/ORM). Multi-scale segmentation has often been linked with hierarchy theory (Hay et al., 2001, Burnett and Blaschke, 2003, Lang, 2008). This association seems obvious as both hierarchy theory and multi-scale segmentation deal with hierarchical organization. As a prerequisite, hierarchy theory proposes the (near-) decomposability of complex systems (Simon, 1973) resulting in “holons” (Koestler, 1967) as hierarchically organized, and multiscale *‘*whole-parts*’*. But as imagery is just a (flat and spectral) representation of such systems, Lang (2008) introduced the term “geons” as proxies for holons. He suggests starting from delineating image regions and then reaching towards image-objects (geons) while applying segmentation and classification routines in a cyclic manner. At first glance, hierarchical segmentation produces regions of increasing average size which need to be linked to organisational levels (Fig. 6, also cf. Fig. 5). The quests for “the right” segmentation level and for “significant” scales has led to dozens of empirical investigations and the development of numerous statistical methods (Hay et al., 2001, Drăguţ et al., 2010).

### 4.7 Objects, ontologies and semantics

To translate spectral characteristics of image objects to real-world features, GEOBIA uses semantics based on descriptive assessment and knowledge, this means, it incorporates “the wisdom of the user”. When studying today*’*s plethora of literature we may reason that access to information content by users is a key success factor at several levels, both *within* one data set and *between* data sets. Commercial data providers and agencies need effective interfaces for image content so that organizations can maximize productivity when working with geospatial data. Users need access to a trusted, up-to-date source of (multiscale) geospatial data that is easy and flexible to use [and which includes a ‘custody-chain’ of supporting metadata (Hay and Castilla, 2008)]. However, the diversity of users, from government agency experts to ordinary citizens, represents a significant challenge for effective information access and dissemination. Indeed, there is no “one size fits all” solution; however, this situation can exactly be the strength of GEOBIA.

Increasingly often, a distinct land-cover class may need to be regarded as a user-driven set of conditions. Such a ‘user-centred cover-class’ may not necessarily be restricted to extractable features, such as single trees – when classifying orchards. Such demand calls for recognized and well defined ontologies in order to avoid stand-alone and black-box solutions (see next sub-section). GEOBIA methods allow for ‘putting groups of pixels into context’ (see Section 4.3). Lang et al. (2009) go one step further and describe conditioned information as the result of a process to fulfil user demands. Geons (Lang, 2008, Lang et al., 2009) are the building blocks of this process of information conditioning, being flexible spatial units, providing a policy-oriented, scaled representation of administered space, but not confined by administrative units (Lang et al., 2010).

Objects may exist as *bona fide* objects or as *fiat* objects (Smith and Varzi, 2000), thus they exist without or with human appreciation (Castilla and Hay, 2008). Intuitively we may think of *bona fide* objects sensu *geographical entities* as the primary target objects of an image analysis task, i.e. entities that can be clearly delineated by human vision and assigned critical spectral or geometrical features (cf. Fig. 4, Fig. 6).

As a branch of philosophy, ontology studies the constituents of reality. An ontology of a given domain describes the constituents of reality within that domain in a systematic way, as well as the relations between these constituents and the relations of these to constituents of other domains. Terms such as ‘domain’, ‘constituent’, ‘reality’ and ‘relation’ are themselves ontological terms, as are ‘feature’, ‘object’, ‘entity’, ‘item’, as well as ‘being’ and ‘existence’ themselves (*ibid*.). Information scientists may use the term ‘ontology’ differently to philosophers. Typically, they designate the regimentation of such conceptualizations through the development of tools designed to render them explicit, such as point, line, and polygon, etc. Geographical Information Systems typically impose simple semantic structure about the world a-priori – mainly a textual metadata description – or leave the semantics to the user. One example may be a forest map: categories could be ‘deciduous’ or “coniferous’ or ‘commercial forest’ which can have totally different meaning in different countries.

**Fig. 7:**
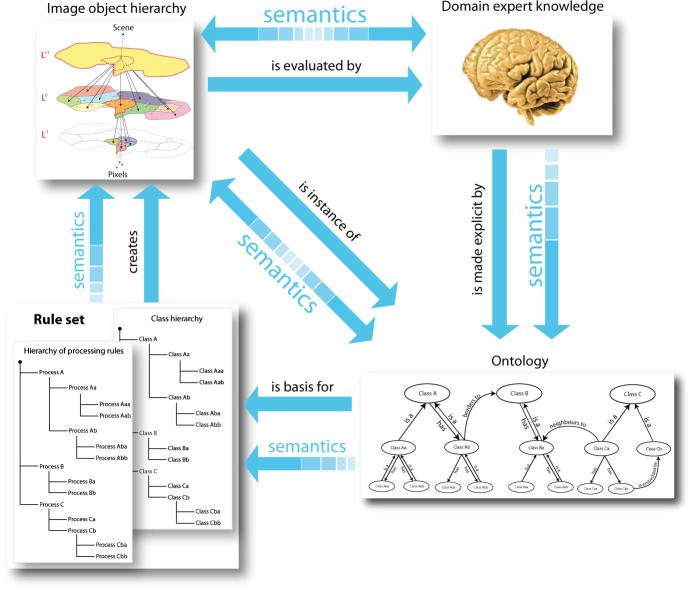
Principle of the iterative workflow in GEOBIA: Initially generated image-objects are classified and enhanced iteratively step-by-step by incorporating procedural and object-domain knowledge described in an ontology and expressed and applied in a rule set.

Smith and Mark (2001) argue for a top-level geographic domain within their *proposed general theory*, with a pertinent basic level category being land cover entities such as mountain, hill, island, lake. Within GEOBIA we can also cope with land-cover categories which are perceivable, yet not easily extractable based solely on internal heterogeneity (so-called modelled composite classes, e.g. an orchard). This links GEOBIA to GIScience from which other concepts of continuous fields, discrete objects, and field objects (Yuan, 2001) need to be adopted. In particular, GIScience may serve as a theoretical underpinning for *object fields* (Cova and Goodchild, 2002) and the *association classes* of the object-oriented approach to data modelling. We claim that GEOBIA concepts support and also require semantics, which we illustrate in Fig. 7.

Until recently, the majority of GEOBIA methods started with the identification of objects such as buildings or trees. Only recently, methods are being developed which start from a combined spatial semantics and thematic semantics approach of feature types, particularly when addressing complex geospatial features. Tiede et al. (2012) establish the implementation of a GEOBIA geoprocessing service. Yue et al. (2013) propose a workflow-based approach for discovery of complex geospatial features that uses geospatial semantics and services. Andres et al. (2012) demonstrate how expert knowledge explanation via ontologies can improve automation of satellite image exploitation by starting from an image ontology for describing image segments based on spectral, pseudo-spectral and textural features. Arvor et al. (2013) comprehensively portray ontologies in GEOBIA, especially for data discovery, automatic image interpretation, data interoperability, workflow management and data publication. This seems to become a now trend in remote sensing and GIScience – namely first conceptualizing real world classes and the starting analysis procedures.

## 5 Conclusions

This article builds the rationale for considering Geographic Object-Based Image Analysis (GEOBIA) as a new and evolving paradigm in remote sensing and to some degree in GIScience. It does so by defining many of the key concepts. GEOBIA is strongly associated with the notion of image segmentation but this article reveals that this is only *one* but very typical geo-object-based delineation strategy. GIS-like functionality is used in classification procedures. This makes GEOBIA context-aware but also multi-source capable. When the methods become contextual they allow for the utilization of ‘surrounding’ information and attributes. This increases the importance of ontologies – as compared to the per-pixel analysis. The workflows are usually highly customizable or adaptive allowing for the inclusion of human semantics and hierarchical networks.

Given the diversity of geospatial data beyond images and the necessity for multidisciplinary research, achieving efficient and accurate data integration is fundamental to the effectiveness of GEOBIA and may become a unique feature of GEOBIA compared to other geospatial approaches. Researchers from biology, geography, geology, hydrology and other disciplines need to access common data sets and combine them with their discipline-specific data. They also need to be able to load and share their thematic layers “intelligently”. Furthermore, GEOBIA has to provide types of models and forms of spatial analysis that are increasingly needed to solve time sensitive social and environmental problems. Thus, an evolving GEOBIA needs to provide solutions to integrate data of widely varied quality, and spatio-temporal scales and resolutions.

We found an increasing number of GEOBIA peer-reviewed publications, special issues, books, commercial and free and open source software, and specific job openings for experienced practitioners etc. and we concluded that GEOBIA is an evolving paradigm. Like other juvenile approaches we may still witness terminological ambiguities. But based on the discussion of underlying principles and methods we are confident that GEOBIA is not just a collection of segmentation, analysis and classification methods. It is an evolving paradigm with specific tools, software, methods, rules, and language, and it is increasingly being used for studies which need to conceptualize and formalize knowledge representing location based reality.

Future research needs to transform GEOBIA databases into more comprehensive (web-enabled) geographic knowledge-bases supporting knowledge discovery and analysis far beyond classic mapping, similar to recent GIS where scientific knowledge is or should increasingly be based on the formalization of geospatial semantics and support for shared knowledge and collective intelligence (Harvey and Raskin, 2011). This will facilitate the exploitation of the enormous amounts of information currently residing in images and image archives, transforming them into web accessible value-added knowledge products. To reach this potential, GEOBIA needs to adopt an appropriate, flexible and robust geospatial digital earth model that allows for the linking/querying of multiscale object attributes, and location traceable neighbourhoods through time and over different mapping projections.
